# Application of an Australian Dietary Guideline Index to Weighed Food Records

**DOI:** 10.3390/nu11061286

**Published:** 2019-06-06

**Authors:** Susan J. Ward, Alison M. Coates, Alison M. Hill

**Affiliations:** 1 School of Health Sciences, City East Campus, University of South Australia, GPO Box 2471, ADELAIDE, SA 5001, Australia; susan.ward@mymail.unisa.edu.au (S.J.W.); alison.coates@unisa.edu.au (A.M.C.); 2 School of Pharmacy and Medical Sciences, City East Campus, University of South Australia, GPO Box 2471, ADELAIDE, SA 5001, Australia

**Keywords:** Dietary Guideline Index, diet quality, weighed food records, adults, Australia

## Abstract

The Dietary Guideline Index (DGI) is a validated diet quality index that reflects adherence to the Australian Dietary Guidelines. The aim of the current study was to establish a novel methodology that applied the DGI to dietary data collected via gold standard, weighed food records (WFR). Consisting of 10 components with a maximal score of 120, the DGI reflected the food-based recommendations of the current Australian Dietary Guidelines and included indicators to score adequacy and quality of core food components and discretionary choices within the diet. The DGI was applied to WFR collected from a sample of 141 adults (84 women, 57 men). Differences between gender for each indicator, as well as subscores for core and noncore components of the DGI were examined. Construct validity was assessed by evaluating the relationship between total DGI score and intake of key nutrients of interest. Overall, the median DGI score was low, 50.87 (range 20.6–104.1). Higher DGI scores were associated with lower intakes of saturated fat, added sugars and sodium (*P* < 0.05). This methodological approach of applying the DGI to WFR may improve our ability to quantify diet quality, thereby providing a tool to assess changes in dietary intake over time and allow the quantification of diet quality as a variable in relation to health outcomes.

## 1. Introduction

Diet is a major determinant of chronic disease risk, impacting morbidity, mortality and quality of life [1]. It is increasingly recognised that it is the synergistic interactions between foods, nutrients and non-nutrient components that exert beneficial health effects or lead to detrimental health outcomes [2]. Assessment of dietary patterns takes into consideration the quantity, proportion, frequency, variety and combinations of beverages and foods consumed [3,4,5,6], and there is increasing consensus among researchers and policy makers that approaches to measure dietary patterns recognise the complexities within whole diets in relation to health outcomes [6]. Scoring of whole diets, over isolated nutrients, allows protective dietary patterns and unfavourable dietary components to be identified [7] and associations with health measures to be made [4,8]. Dietary patterns that promote health and reduce risk of chronic disease are captured within evidence-based dietary guidelines, with individuals who adhere to dietary guidelines proposed to have higher diet quality [2].

The Dietary Guideline Index (DGI) is a diet quality index initially developed by McNaughton et al. [9] to evaluate adherence to the Australian Dietary Guidelines. Primarily, the DGI has been scored using dietary data collected from semiquantitative food frequency questionnaires (FFQ) with additional food habits questions [9,10]. The DGI has been revised to reflect updated guidelines [10], adapted for use with 24-hour recall data in children [11] and adults [12,13], and has been validated against nutrient intake [9,11]. DGI scores demonstrate expected associations with sociodemographic factors and health behaviours, with higher DGI scores associated with self-assessed health status, reduced energy consumption, lower body mass index (BMI) and lower risk of hypertension and type 2 diabetes [9,10,14]. To date, this index has not been applied to data collected from weighed food records (WFR), which are considered the gold standard of dietary data collection [15,16]. Therefore, the aim of this study was to adapt the design and scoring of the DGI to WFR to produce a measure of diet quality.

## 2. Materials and Methods

The process for revising the structure of the DGI included updating of components to reflect food-based recommendations of the 2013 Australian Dietary Guidelines [17] (Table 1). Consistent with previous studies [9,10], for each component, indicators and scoring were guided by age and specific recommendations for men and women as outlined in the Australian Guide to Healthy Eating (AGHE) [18]. Once the structure of the DGI was finalised, it was applied to baseline data collected from a sample of middle-aged to older Australian adults (*n* = 141) who had participated in a randomised controlled dietary intervention trial.

### 2.1. Development of the DGI Components

To reflect the current food-based dietary guidelines, components and indicators of the DGI were developed in line with Australian Dietary Guidelines recommendations on quantity and quality of core and noncore food and beverages. The DGI for this study, therefore, consisted of ten components with 12 indicators (Table 1). Seven core-food components reflect adequacy and quality of intake within the Australian Dietary Guidelines core food groups; one component for variety of intake of core-foods, five components for each of the core food groups (vegetables, fruit, grains, lean meats and alternatives, and dairy and alternatives), and one for water intake. Within the core-food components, subcomponents reflecting quality of food choices, as recommended in the Australian Dietary Guidelines, were included for grain and dairy foods, and for fluid intake. The three noncore food components included an allowance for unsaturated spreads or oils, discretionary items and alcohol, reflecting compliance with guidelines to moderate or limit intake.

### 2.2. Development and Scoring of Specific Indicators for Each DGI Component

#### 2.2.1. Core Food Components

Within the core food groups, variety of intake was captured using the Australian Recommended Food Score (ARFS) [23]. The core food group components for vegetables, fruits, lean meat and alternatives were based on the number of serves per day consumed (one indicator each). Grain and dairy components were separated into two indicators, each reflecting adequacy (serves per day) and the quality of food choices within these components. Water intake also included two indicators reflecting usual fluid consumption and the preference for fluids to come predominantly from water.

In the development of the index, there were several decisions regarding criteria for indicators that required addressing. Lean meats and alternatives did not include processed or discretionary meats (i.e., ham, bacon, salami and poultry luncheon meats); it included serves of lean meat, poultry and fish, eggs, nuts, seeds and tofu. The Australian Dietary Guidelines places processed and cured meats in the ‘discretionary foods’ category due to their content of saturated fat and added salt and links to cancer outcomes [17]. To be consistent with the Australian Dietary Guidelines, lean and unprocessed meats contained <10% total fat. In addition, we excluded those meats listed in the Discretionary Food List established for the Australian Health Survey which included battered or deep-fried sources [17,24]. All soy products were assigned to lean meats and alternatives, except for soymilk, which was included in the dairy and alternatives component as a dairy substitute. Dairy serves included plain milks and predominantly milk-based drinks such as flavoured milks and espresso-based coffee drinks (e.g., flat white, latte). It also included hard and soft cheeses, dairy desserts (such as custards) and calcium-fortified dairy substitutes.

The impact of including (up to a maximum of one serve) 100% fruit juice or dried fruit as a fruit serve was considered. Several diet quality indices [10,13,25,26] have excluded these higher sugar content fruit alternatives from fruit intake. In line with the current Australian guidelines that they may occasionally be substituted for other fruits [17], the fruit component included 100% fruit juice (not fruit drinks) or dried fruit up to 1 serve per day. Vegetable serves included legumes and beans.

Grain, dairy and fluid components were each separated into two indicators reflecting adequacy (serves per day) and the quality of food choices within these components. The wholegrains component captured choosing nutrient-dense and high fibre wholegrain bread and cereal foods rather than less nutrient-dense refined grain and cereal products, and the reduced/low-fat dairy component reflected meeting nutrient requirements within energy requirements [17]. Total beverage intake reflected nondiscretionary sources of fluid and included water, 100% fruit juice, diet soft drinks and cordials, unsweetened tea and coffee, and milk and milk-based beverages.

#### 2.2.2. Core Component Scoring

Components were each scored out of 10; achieving maximal scores indicated full compliance with guideline recommendations. Total scores for core-food components ranged from 0–70, with higher scores reflecting greater adherence with the Australian Dietary Guidelines and therefore a better-quality diet.

The indicator for dietary variety was scored based on the Australian Recommended Food Score, which awards points for reported frequency of consumption of different varieties of foods/beverages within the core-food groups [23]. Subscales for reported intake of ≥once per week of different vegetables, fruits, grains, meats and alternatives and dairy and alternatives were summed to give a score for variety.

For total daily intake of vegetables, fruit, lean meats and alternatives, a maximal score indicated full compliance with guideline-recommended serves and a zero-score indicated that minimum guidelines were not being met. Proportionate scores were awarded for number of serves falling between cut-offs. For example, the recommended number of serves of vegetables for 50–70-year-old males is 5.5 serves per day, and a man achieving this would achieve a maximal score of 10. A man having no serves of vegetables would achieve a zero score, while consuming between 0 and 5.5 serves would achieve a proportionate score. Thus, a man consuming 4 serves of vegetables, but needing to consume 5.5 serves, would be scored as (4/5.5) × 10 = 7.3.

The grain and dairy components were also scored out of 10; five points were allocated to the number of serves per day (adequacy) and five points attributed to the quality of food choices within these components (proportion of wholegrain and reduced fat dairy). Proportionate scores (described above) were awarded for number of serves, whereas indicators scoring quality of grain and dairy intake had only minimal or maximal scores based on whether indicators were met/not met. The two indicators for fluid intake were scored similarly (five points each), with proportionate scores for the number of beverages consumed based on a maximal score of 8–10 cups per day. For the proportion of water to total beverages, a maximal score was awarded if more than 50% of total fluid intake came from water. For intake less than 50%, a proportional score was calculated.

#### 2.2.3. Noncore Food Components

Three indicators reflected the noncore food components to moderate or limit intake. Firstly, the component for unsaturated spreads or oils recognised a moderate intake of unsaturated fats from plant sources, avocado, nut and seed pastes (not whole nuts) supplies essential nutrients and contributes to health when replacing dietary saturated fatty acids [17]; however, unsaturated spreads or oils should be consumed in moderation due to their high energy content. Secondly, discretionary choices are defined as being foods and drinks high in saturated fat and/or added sugars or salt and, as reflected in the DGI, they are not recommended as part of the diet for adults above their healthy weight range [17]. Thirdly, alcohol is considered part of this category to limit intake due to lack of essential nutrients and its associated health risks. In line with the National Health and Medical Research Council Alcohol Guidelines [22], standard drink measures (10 g alcohol) were used to determine serves of alcohol rather than considering alcohol’s contribution to energy.

#### 2.2.4. Noncore Component Scoring

Intake of the noncore food components were scored to reflect the moderation or limiting of intake. Total scores for the noncore food components ranged from 0–50; 10 points each for allowance of unsaturated fats and alcohol, and 30 points for discretionary foods. The allowance for unsaturated spreads and oils is structured as a range of permitted amounts [17]; this component awarded maximal scores if serves fell within this range and zero points if outside this range. Alcohol was awarded either maximal or minimal scores for meeting or not meeting the scoring criteria. Discretionary items were reverse-scored, with a maximal score of 30 points being awarded for consuming between zero and 2.5 or 3 discretionary serves for women and men, respectively. A zero score was applied to persons consuming more than these amounts. The higher weighting allocated to the discretionary component of the DGI relative to other components (i.e., 30 points vs. 10 points) addresses recent concerns about the weighting of discretionary items with regard to diet quality index scores [27,28]. Furthermore, in order to keep this a food-based index, this scoring allowed for some components from the original DGI [9,10] referring to nutrients to limit (saturated fat, sodium and added sugars) to be encompassed into the indicator to limit discretionary foods.

Total scores for the DGI therefore ranged from 0 to 120, with scores for core-food components from 0–70 and noncore food components from 0–50.

### 2.3. Application of the DGI

#### 2.3.1. Population

The DGI was applied to a sample of 141 participants with complete food diaries enrolled in a randomised controlled parallel arm dietary intervention trial conducted at the University of South Australia. Written informed consent was obtained from all participants, with the main intervention study approved by the University of South Australian Human Research Ethics Committee and registered on the Australian New Zealand Clinical Trials Registry (ANZCTR) (ACTRN12615001294549). Participants were middle-aged to older (50 to 80 years), overweight or obese (BMI 25–39.9 kg/m^2^) nonsmokers and free of established cardiovascular, neurological or diabetic conditions.

#### 2.3.2. Anthropometric Data

Participants’ weight and height measurements were taken twice and averaged to calculate BMI to the nearest 0.1 kg/m^2^. Weight was measured to the nearest 0.1 kg using TANITA Ultimate Scale 2000 (Tanita Corporation, Tokyo, Japan) and height measured to the nearest 0.1 cm using a wall-mounted stadiometer (SECA; Vogel & Halke, Hamburg, Germany).

#### 2.3.3. Dietary Intake

Dietary intake was assessed using 3-day WFR. Weighed food records are considered the gold standard dietary data collection method in determining nutrient and energy intake in free living individuals [15,16]. Intake was reported on consecutive days; 2 weekdays and 1 weekend day, as higher energy intake is known to be reported on weekends [16]. The use of WFR allowed detailed descriptions of food intake, packaged foods, recipes and cooking methods.

Scales and a booklet with detailed instructions to record weights or volumes of all foods and drinks consumed were provided to participants. Participants were instructed to report the timing of food intake and to record recipes, brands or packaging from ready-made meals or store-bought products, as well as food preparation and cooking methods. When scales were unable to be used, such as when dining out, participants were instructed to use common household measures to estimate quantities. Weighed food records were reviewed for completeness and entered into FoodWorks Nutritional Analysis Software Version 8 (Xyris Software, Australia) for analysis (which was the most recent version at time of entry), and nutrient data were derived from the Australian Food and Nutrient (AUSNUT) 2011-13 [29] food composition database. Data were obtained for energy, macro- and micronutrient intake. Established cut-offs of <4000 kJ or >17000 kJ/day were applied to total energy intake to exclude participants suspected of under- or overestimating daily intake [30].

#### 2.3.4. Calculation of Food Serves

FoodWorks utilises the AUSNUT 2011-13 food composition database [29] to code and classify foods and beverages based on the Australian Dietary Guidelines five core food groups (vegetables, fruit, grains, lean meat and alternatives, and dairy), with additional categories for water and unsaturated spreads and oils [17]. The numbers of serves for core food groups were established using standard serving sizes from the Australian Guide to Healthy Eating as provided through FoodWorks. Total serves were determined for each DGI core food group component from reported intake of individual foods, as well as composite foods and mixed dishes. The AUSNUT 2011-13 database recipe file [31] disaggregates mixed dishes to enable the classification and assignment of components of mixed dishes to their respective core food groups (for example a stir fry would be broken down to amounts of meat, vegetables, grains and oils). The disaggregation of mixed dishes into their food groups allowed for a more accurate representation of food group intake.

Within the Australian Guide to Healthy Eating, discretionary foods are considered as whole foods as this is how they are consumed; however, FoodWorks does not specifically identify discretionary foods and beverages and instead breaks these down and assigns relevant components to core food groups. For example, an apple pie is distributed among the grain and fruit groups, along with estimates for added sugars and solid fats. Therefore, to provide a serve estimate, discretionary foods and beverages were first identified using the discretionary food list established for the Australian Health Survey [32]. Serving size was then calculated by dividing the total energy for that discretionary food or beverage by 600kJ, which is the energy value attributed to one serve of a discretionary item in the Australian Guide to Healthy Eating [18].

#### 2.3.5. Age- and Gender-Specific Cut-offs

Data from reported intakes over the 3 days of the WFR were averaged to establish daily serves and, from these, allocate scores for each indicator of the DGI. Scoring consisted of comparing intake against the recommended serves per day specific to age and gender, with points being awarded for the number of serves consumed up to the recommendations (awarded the maximal score). As detailed in Table 1, scoring cut-offs for indicators were based on Australian Dietary Guidelines age and specific serves per day for men and women 51 to 70 years, and over 70 years [17]. Rather than include a separate category for participants aged 50 years in this study, the cut-offs pertaining to 51–70 years were extended to 50 years to capture the age range of study participants.

### 2.4. Analysis

Statistical analyses were undertaken using SPSS 25.0 (IBM, Armonk, New York). Data were checked for completeness and normality prior to analysis. For all analyses, statistical significance was set at *p* < 0.05. Gender differences in participant characteristics (age, height, weight, BMI and energy intake) were determined using independent t-tests for normally distributed data and Mann–Whitney U tests for non-normally distributed variables.

Scores for individual indicators along with the proportion of participants meeting the guidelines (achieving maximal scores for the indicator) were calculated. Mann–Whitney U tests were used to determine gender differences in components and chi square tests to compare proportions complying with guidelines. If variables were not normally distributed, values were reported as median and interquartile range (IQR); dichotomous components (i.e., meeting or not meeting) are reported as median only. Construct validity of the DGI (ability of the DGI to capture nutrient intake) was evaluated by correlating total DGI scores with intake of key individual nutrients, with and without adjusting for energy intake. DGI scores were log transformed prior to analysis, and the strength of associations was determined using Pearson correlations.

## 3. Results

Participant characteristics are presented in Table 2. A total of 141 participants were included in results. Women were significantly younger, shorter and lighter than men, but there were no gender differences in BMI. All participants fell within the guidelines for plausible energy intake.

Total DGI scores for the whole group ranged from 20.6 to 104.1, with a median score of 50.9 (IQR 19.1) out of a maximum score of 120 (Table 3). Median total DGI score was higher for women than men (*P* < 0.05), as was the total core food components score (*P* = 0.001) of the DGI. Women scored significantly higher than men within the core food components for food variety and intake of grains, lean meats and fluids, and within the noncore components for limiting alcohol intake. Men and women both scored lowest on the components to limit discretionary intake and moderating intake of unsaturated spreads and oils. The components for intake and quality of dairy were the next lowest scoring components for both men and women.

The proportion of participants achieving a full score for any of the DGI components was low. No participants met recommendations for food variety, and few participants met guidelines for vegetable intake (10.6%). Compliance with guidelines for intake of dairy and alternatives among participants was low (5.7%), as was the proportion choosing reduced fat dairy (14.9%). The greatest compliance with recommendations was seen in the component to limit alcohol intake, with 92% of women and 77% of men achieving this guideline and this being the highest-scoring component. Few participants (17%) met the guideline to limit discretionary intake; the reported average number of serves of discretionary items for the 3 days intake ranged from 0.25 to 14.25 serves, with median serves being 4.80 (IQR 3.39). Discretionary serves were not statistically different between women and men (4.66 (IQR 2.62) and 5.74 (IQR 3.83) serves, respectively; *P* = 0.055).

### Association with Energy and Nutrient Intake

Higher total DGI scores were associated with lower energy intakes (*r* = −0187, P-trend = 0.026), but not BMI (*r* = −0.012, P-trend = 0.884).

A higher DGI score (energy adjusted) was significantly associated with lower intakes of saturated fat, added sugars and sodium. Higher DGI scores were associated with higher intakes of protein, total sugars, dietary fibre, calcium, iron, zinc, potassium, magnesium, iodine, vitamin A equivalents, β-carotene equivalents, total folate, vitamin B-12, riboflavin, vitamin C, vitamin E and α-tocopherol (Table 4).

## 4. Discussion

This paper describes the development and application of a DGI to WFR and demonstrates its capability as a tool to measure diet quality and capture adherence to the current food-based Australian Dietary Guidelines. In this sample of mid- to older age overweight and obese adults, adherence to the current Australian Dietary Guidelines was poor, a finding consistent with previous studies using this index with FFQ [9,10]. Similarly, we also observed higher diet quality in women compared with men [4,9,10,12,33], though both women and men showed suboptimal compliance across both core and noncore food components. Less than half of participants met guidelines for intake of fruit, vegetables, lean meat and alternatives, and dairy and alternatives and no participant met guidelines for variety of intake. Less than 20% of participants met guidelines for limiting the intake of discretionary items [10,33].

There is the perception that individuals with high energy consumption are more likely to achieve cut-offs for food group serves, thus potentially obtaining an overall higher diet quality score [28]. This DGI did not adjust for energy intake, or account for excessive intakes within individual food groups; however, the upper limit to scores avoids rewarding intake beyond recommendations with additional points or a higher than maximal score. Additionally, excess consumption of typically energy-dense discretionary foods was not rewarded. This approach is consistent with other diet quality indices and avoids penalising high consumption of core foods [10,27,28,34]. Higher diet quality in this study was associated with reduced energy intake, which indicated the DGI’s ability to discriminate higher quality diets through meeting guideline recommendations rather than overconsumption. Discretionary foods are major contributors to energy intake and to nutrition-related burden of disease [17,33]. Inclusion of these foods in the diet is an indicator of poor diet quality as they displace nutrient-dense foods from the diet [2]. A key finding of the Australian Health Survey 2011–2012 was that Australians obtain a high proportion (35%) of their daily energy requirements from discretionary sources [33]. Findings in the current study support this, with over 75% of participants consuming more than 3 serves of discretionary foods a day and 31% reporting consuming more than 6 discretionary serves a day. Discretionary foods contributed on average 3200kJ/day or 36% of the average daily energy intake (~9000kJ/day). It is important to note that intake of discretionary items is not recommended for individuals who are above their healthy weight [21]. The scoring of discretionary items in this study was consistent with other DGI in that it allowed some discretionary items to be consumed irrespective of weight status (0–2.5 or 0–3 serves for women and men, respectively). Had we allocated a threshold of zero serves per day to achieve maximal scoring for this component, then all the overweight participants in this study would have received zero points for this component. Interestingly, when we reran correlation analyses with these adjusted DGI scores against nutrients of interest, the relationships remained largely unchanged (data not shown). A recent systematic review reported that diet quality indices based on dietary guidelines were consistently inversely associated with measures of weight status [35]. The lack of association between DGI scores and BMI in this study is inconsistent with this and findings from other studies applying the DGI [4,9,10,12,36,37]; however, as participants within our sample were either overweight or obese, this may be due to the limited spread of data.

With ageing, requirements of some nutrients increase while overall energy requirements decrease, emphasising the importance of choosing nutrient-dense foods as promoted by dietary guidelines [17]. In this study, we observed moderate associations between diet quality score, reflecting adherence to national dietary guidelines, and increased intake of key nutrients of interest; protein, calcium, zinc, vitamin A equivalents, β-carotene equivalents, riboflavin, vitamin C and vitamin E. The strongest relationships were seen for dietary fibre, potassium and magnesium intake.

Higher DGI scores also were moderately associated with lower intakes of added sugars, total, saturated and monounsaturated fat, though relationships further weakened when adjusting for energy intake. Although statistically significant, higher DGI scores were only weakly associated with lower sodium intake, possibly reflecting the contribution of core foods such as grains and dairy to sodium intake. The positive association with total sugars is reflective of all sugars found in the diet, including those found in fruit and dairy; therefore, associations with added sugars are more relevant from a diet quality perspective. The strength and breadth of significant associations observed in this study demonstrate the indices’ ability to reflect nutrient intake even though components were based on food-based dietary guidelines, rather than a reductionist nutrient-centric approach.

Weighed food records allow researchers to obtain detailed qualitative and quantitative dietary information [38,39]. Use of this dietary collection method enabled us to define criteria for inclusion of certain foods within each component, which is a distinct advantage over other DGIs. This was particularly relevant for capturing quality of meats, grains and dairy. Qualification of cuts of meat, along with cooking method and degree of processing, better reflected recommendations to choose lean meats. Scoring of wholegrain or low-fat dairy in other DGIs with FFQs has been limited to identifying the type of bread or milk usually consumed. The subcomponent capturing the quality of grains was expanded to capture the proportion of all higher-fibre forms of grain or cereal foods reported, such as oats and wholegrain versions of pastas, noodles and brown rice. Similarly, to reflect intake of nutrient-dense dairy serves, the component to choose reduced-fat dairy captured serves of low- or reduced-fat dairy foods. Additionally, we recognised the contribution of small quantities of unsaturated oils, fats and spreads to health by awarding a maximal score if participants fell within the recommended range [17]. This is different to other scores that award points below a specific cut-off, which means that participants who do not consume any healthy fats are given a maximal score [9,10].

It has previously been emphasised that the inclusion of both healthy and unhealthy components is important where providing specific feedback to individuals or target populations regarding compliance with the guidelines [27]. Being structured as a food-based index, this DGI provides the ability to identify which specific components should be modified to better achieve adherence to dietary guidelines. However, the cross-sectional nature of the current application limits this study’s ability to infer causality, where changing intake of foods may influence diet quality and/or health outcomes. Applying the DGI to longitudinal data and evaluating associated changes in health outcomes is an important next step. Furthermore, generalisability of results was restricted by the number of participants, and homogeneity within the group with respect to age, ethnicity, and BMI limiting comparisons to other studies in this age group. Performance of this DGI needs to be assessed in a larger population of varying ages, weight status, ethnicity and socioeconomic status. Dietary data from only 3 days limited the ability to fully capture habitual intake and dietary variety. Dietary variety is important in optimising nutrient intake and is associated with improved quality of life and reduced risk of chronic disease and mortality [14,17]. It should also be noted that whilst the dietary data collected on consecutive days may be correlated and may therefore influence the diet quality score for an individual, this should not have affected the ability of the DGI to assess diet quality. This study followed the Discretionary Food List [24], which meant that some dessert-style dairy foods containing higher proportions of added sugar and saturated fat (for example, custard) were included in the dairy and alternatives component rather than being considered discretionary items. Similarly, oil-roasted and salted nuts were included in the meat and nonmeat alternatives component, as they were not flagged as discretionary items in the Discretionary Food List [24]. The DGI also did not distinguish between types of meat or fish eaten within the lean meats and alternatives component (e.g., beef versus tuna). In a recent meta-analysis of food groups and risk of all-cause mortality [40], consumption of fish was inversely associated with all-cause mortality, whereas a positive association was seen with red meat.

## 5. Conclusions

This study builds on existing diet quality indices for Australian adults through the development of an index based on the evaluation of diets captured via WFR. This approach strengthens our ability to capture quantity, quality and variety within the complexity of whole diets. Furthermore, the food-based scoring methodology assists in identifying areas that require improvement, both in terms of quality and quantity, which will facilitate the delivery of tailored nutrition advice. The DGI presented here had moderate associations with key nutrients, suggesting good construct validity, with higher scores reflecting a nutrient-rich dietary pattern. Although further work is needed to investigate its reproducibility and use in more diverse populations, this DGI has applications for longitudinal epidemiological or clinical studies assessing usual intakes or investigating changes in consumption patterns in relation to dietary intervention or other health-related behaviours, which may improve health outcomes.

## Figures and Tables

**Table 1:** Components and scoring approach for the Dietary Guideline Index to measure diet quality in middle-aged to older Australian adults who participated in a dietary intervention trial ^1^.

Dietary Guideline	Indicator & Description	Criteria for Minimum Score		Criteria for Maximum Score		Component Score
				50–70 ^2^ years	≥70 years	
**Core food components: adequate intake**						
1. Enjoy a wide variety of nutritious foods from each of the 5 core food groups every day	Food variety: proportion of food types from each of the 5 core food groups consumed at least once per week	0%		100%		10
2. Plenty of vegetables, legumes/beans	Total vegetable intake: serves of vegetables per day	0		M ≥ 5.5 W ≥ 5	M ≥ 5 W ≥ 5	10
3. Fruit	Total fruit intake: serves of fruit per day	0		M ≥ 2 W ≥ 2		10
4. Grain (cereal) foods	Total grain intake: serves of grains per day	0		M ≥ 6 W ≥ 4	M ≥ 4.5 W ≥ 3	5
	4a Mostly wholegrain or high fibre cereals: serves of wholegrain as a proportion of total grains	<50%		≥50%		5
5. Lean meat and poultry, fish, eggs, nuts and seeds, and legumes/beans	Total lean meat and alternatives: serves per day (excludes processed meat)	0		M ≥ 2.5 W ≥ 2		10
6. Milk, yoghurt, cheese and/or their alternatives	Total dairy and alternatives: serves per day	0		M ≥ 2.5 W ≥ 4	M ≥ 3.5 W ≥ 4	5
	6a. Choose reduced-fat: type of dairy usually consumed	<100%		100%		5
7. Drink plenty of water ^3^	Total beverage intake: serves per day	0		M ≥ 10 W ≥ 8		5
	7a. Proportion of water consumed relative to total mg of beverages ^4^	0%		≥50%		5
Subtotal: Core food components						70
**Noncore food components: moderate or limit intake**						
		50–70 years	≥70 years			
8. Small allowance of unsaturated oils, fats or spreads ^5^	Unsaturated spreads or oils: serves per day	M < 1 or > 4 W < 1 or > 2	M & W < 1 or > 2	M 1–4 W 1–2	M 1–2 W 1–2	10
9. Limit intake of foods and drinks containing saturated fat, added salt and added sugars ^6^	Limit discretionary foods: serves of discretionary foods per day	M > 3 W > 2.5		M ≤ 3 W ≤ 2.5		30
10. If you choose to drink alcohol, limit intake ^7^	Limit alcohol: serves per day	>2		≤2		10
Subtotal: Noncore food components						50
Total DGI Score						120

^1^ Adapted from Thorpe et al. [10]; ^2^ the Australian Dietary Guidelines [17] cut-off pertaining to men (M) and women (W) aged 51–70 years was extended to 50 years to capture the age range of study participants; ^3^ quantitative guidelines derived from Nutrient Reference Values for Australia and New Zealand [19]; ^4^ proportion of water to total beverage consumption based on US beverage consumption [10,20]; ^5^ moderate consumption of poly- and monounsaturated fats supplies essential nutrients and is an important part of the diet [17]; ^6^ recommended ranges for discretionary items are for people who are of a healthy weight [21]; ^7^ National Health and Medical Research Council alcohol guidelines for standard drinks (10 g alcohol/serve) used for cut-offs [22].

**Table 2:** Participant characteristics of 141 middle-aged to older Australian adults at baseline of a dietary intervention trial ^1^.

	Women (*N* = 84)	Men (*N* = 57)	Participants (*N* = 141)
Age (years)	63.2 ± 8.1 *	66.9 ± 7.7	64.7 ± 8.1 ^1^
Height (m)	1.61 ± 0.06 **	1.76 ± 0.7	1.67 ± 0.1
Weight (kg)	79.7 ± 11.2 **	92.5 ± 12.0	84.8 ± 13.1
BMI (kg/m^2^)	30.9 ± 3.8	29.7 ± 3.2	30.4 ± 3.6
Energy Intake (kJ/day)	8762 ± 1894 *	9386 ± 2226	9014 ± 2050

^1^ Values are mean ± standard deviation (SD); * different from men, *P* < 0.05; ** different from men, *P* < 0.01; BMI: body mass index.

**Table 3:** Median scores and IQR for Dietary Guideline Index components, with proportion meeting Australian Dietary Guideline recommendations.

	DGI Component Scores ^1^				Proportion Meeting Guideline ^2^ (%)			
	Total	Women	Men	*P* Value ^3^	Total	Women	Men	*P* Value ^3^
Core food components: adequate intake								
1. Food variety	3.14 (1.08)	3.37 (1.07)	2.88 (1.07)	<0.001	0.0	0.0	0.0	-
2. Vegetables	5.12 (4.17)	5.41 (3.91)	4.59 (4.43)	0.626	10.6	9.50	12.3	0.602
3. Fruit	5.77 (6.72)	6.61 (6.40)	4.63 (7.54)	0.158	29.1	28.6	29.8	0.872
4. Grain (cereal)	5.00 (1.35)	5.00 (0.95)	4.44 (1.91)	0.002	53.9	64.3	38.6	0.003
4a. Mostly wholegrain	0.00	2.50	0.00	0.261	46.1	50.0	40.4	0.259
5. Lean meats and alternatives	5.72 (4.81)	6.24 (4.73)	4.38 (4.11)	0.026	12.8	14.3	10.5	0.512
6. Dairy and alternatives	2.37 (1.52)	2.31 (1.43)	2.55 (1.74)	0.058	5.7	3.6	8.8	0.190
6a. Reduced-fat dairy	0.00	0.00	0.00	0.814	14.9	15.5	14.0	0.814
7. Fluid intake	3.87 (2.27)	4.36 (1.73)	2.83 (2.03)	<0.001	25.5	33.3	14.0	0.010
7a. Proportion water to total fluid	4.23 (2.96)	4.62 (1.98)	3.26 (3.77)	0.067	39.7	41.7	36.8	0.566
Total core food components ^4^	36.72 (13.27)	39.20 (10.83)	32.76 (15.40)	0.001	0.0	0.0	0.0	-
Noncore food components: moderate or limit intake								
8. Moderate unsaturated spreads and oils	0.00	0.00	0.00	0.620	17.7	19.0	15.8	0.619
9. Limit discretionary intake	0.00	0.00	0.00	0.892	17.0	16.7	17.5	0.892
10. Limit alcohol	10.00	10.00	10.00	0.008	85.8	91.7	77.2	0.016
Total noncore food components ^5^	10.00	10.00	10.00	0.224	1.4	2.4	0.0	0.241
Total DGI ^6^	50.87 (19.13)	53.31 (16.61)	45.05 (22.41)	0.008	0.0	0.0	0.0	-

^1^ Data are median (IQR) or median for dichotomous components (no. 4a, 6a, 8–10); ^2^ meeting guideline if attained maximal DGI component score; ^3^ Mann–Whitney U tests were used to determine significant differences between men and women; ^4^ total core food components score range 0–70; ^5^ total noncore food components score range 0–50; ^6^ total DGI scores range 0–120; IQR, interquartile range; DGI, Dietary Guideline Index.

**Table 4:** Correlation coefficients of log transformed Dietary Guideline Index score and nutrients among 141 middle-aged to older Australian adults who participated in a dietary intervention trial.

		DGI Score	
Nutrient of Interest	Median Nutrient Intake (IQR) ^1^	Pearson Correlation (P-Trend)	Partial Correlation (Controlling for EI) (P-Trend)
Protein (g)	91.1 (30.0)	0.102 (0.230)	0.301 (0.000)
Total Fat (g)	80.0 (30.6)	−0.223 (0.008)	−0.123 (0.147)
SFA (g)	31.4 (12.0)	−0.292 (0.000)	−0.244 (0.004)
MUFA (g)	29.4 (13.5)	−0.203 (0.016)	−0.100 (0.240)
VLC-N3 (g)	0.19 (0.43)	0.103 (0.223)	0.108 (0.202)
Sugars (g)	103.6 (44.1)	0.049 (0.562)	0.256 (0.002)
Added sugars (tsp)	7.86 (8.79)	−0.373 (0.000)	−0.329 (0.000)
Dietary fibre (g)	26.3 (11.6)	0.407 (0.000)	0.550 (0.000)
Sodium (mg)	2288.5 (1121.5)	−0.252 (0.003)	−0.193 (0.022)
Calcium (mg)	939.6 (490.8)	0.253 (0.002)	0.375 (0.000)
Iron (mg)	12.0 (4.88)	0.123 (0.148)	0.254 (0.002)
Zinc (mg)	10.7 (4.23)	0.172 (0.042)	0.302 (0.000)
Potassium (mg)	3325.4 (1067.3)	0.389 (0.000)	0.586 (0.000)
Magnesium (mg)	383.2 (141.8)	0.335 (0.000)	0.516 (0.000)
Iodine (µg)	185.5 (76.5)	0.096 (0.259)	0.219 (0.009)
Vitamin A equiv (µg)	947.3 (681.1)	0.297 (0.000)	0.352 (0.000)
Retinol (µg) ^2^	323.9 (231.4)	−0.108 (0.202)	−0.022 (0.793)
β–carotene equiv (µg)	3763.7 (4001.3)	0.436 (0.000)	0.455 (0.000)
Total folate (µg)	544.7 (272.2)	0.165 (0.050)	0.276 (0.001)
Vitamin B-12 (µg) ^2^	4.52 (2.66)	0.154 (0.070)	0.252 (0.003)
Thiamin (mg)	1.57 (0.89)	0.139 (0.101)	0.161 (0.057)
Riboflavin (mg)	2.14 (1.05)	0.268 (0.001)	0.376 (0.000)
Vitamin C (mg)	99.6 (95.7)	0.376 (0.000)	0.405 (0.000)
Vitamin E (mg)	12.5 (5.54)	0.188 (0.026)	0.306 (0.000)
α – tocopherol (mg)	11.3 (4.40)	0.137 (0.104)	0.282 (0.001)

^1^ Data are median (IQR); ^2^ one extreme outlier (more than 3 SD away from the mean) was removed from the analysis of retinol and vitamin B-12. DGI, Dietary Guideline Index; EI: energy intake; IQR, interquartile range; MUFA: monounsaturated fatty acids; SFA: saturated fatty acids; VLC-N3: very long chain omega 3 fatty acids.
